# Dosimetric Advantages of Silicone-Filled Vaginal Spacers in Pediatric Proton Therapy

**DOI:** 10.14338/IJPT-21-00044.1

**Published:** 2022-05-06

**Authors:** Ozgur Ates, Li Zhao, David Sobczak, Chia-ho Hua, Matthew J. Krasin

**Affiliations:** Department of Radiation Oncology, St. Jude Children's Research Hospital, Memphis, TN, USA

**Keywords:** pediatric proton therapy, silicone-filled vaginal spacer, silicone in proton therapy

## Abstract

We introduce a custom-made silicone-filled vaginal spacer for use during treatment of female patients receiving pelvic proton radiation therapy. Commercially available vaginal dilators can be purchased as hollow objects; when filled with a media, they can act as a beam stopper and/or tissue spacer while pushing uninvolved vaginal wall away from a high-dose region. Dosimetric advantages of these specifically constructed silicone-filled vaginal spacers were investigated when compared to the unaltered commercially available product or no vaginal spacer in pediatric proton therapy.

## Introduction

Tissue spacers, including vaginal wall spacers, are used in a number of cancer diagnoses including soft tissue sarcomas, prostate cancer, cervical cancers, and uterine cancer [1, 2]. Treatment of women and children with pelvic radiation therapy can result in vaginal adhesions and scarring that impact function in female genitalia [3, 4]. Efforts to minimize these effects include the use of vaginal wall spacer, either in the form of an insertable plastic device or vaseline-embedded gauze, both designed to move the uninvolved vaginal wall away from the highest doses of radiation in the disease site. The use of a rigid vaginal spacer can be used to provide this necessary reproducible spacing between vaginal walls. The most successful method of reducing radiation late effects is complete avoidance of the tissue at risk by the radiation beams.

Specific to proton therapy, proton beam radiation allows control of the radiation dose by controlling the depth to which radiation will travel. Use of a device such as a vaginal spacer that further moves tissue out of the beam path may even allow better normal tissue radiation protection and possible reduction in late effects. The filling of a commercially available hollow vaginal dilator with a tissue-equivalent material, such as silicone, can allow the device to serve as a beam stopper and provide dosimetric range shifting out of normal healthy tissue. This approach has been studied in other pelvic and abdominal proton radiation treatments, including prostate cancer and retroperitoneal sarcomas [5, 6], along with the physical characteristics and stopping power of these types of material [7, 8].

## Patients and Methods

### Patient Selection and Imaging

Two female adolescents, ages 15 and 16 years, were diagnosed with rhabdomyosarcoma and enrolled in our internal review board–approved multi-institutional protocol for rhabdomyosarcoma. They received proton beam radiation while a silicone-filled vaginal spacer was placed intravaginally on a daily basis during treatment in 2020 to displace normal uninvolved tissue away from the beam path. Patients were imaged twice for treatment planning with and without hollow vaginal spacer, using clinical abdomen protocol in Philips IQon Spectral CT (Amsterdam, Netherlands). The scan parameters were 120 kVp, auto collimation, 500-mm field of view, 512 × 512 matrix size, Standard (B) reconstruction kernel, and dose right index of 20.

### Fabrication of Silicone-Filled Vaginal Spacer

Hollow vaginal dilators (Sarasota, FL) made from medical grade plastic were used to provide tissue spacing. The commercially available dilators came in 5 different sizes ranging from 1.46 cm to 3.65 cm in diameter. An appropriate spacer was selected on the basis of patient size and tolerance, while maximizing spacing of uninvolved tissues. The filling material was chosen to be silicone with a density of 1.15 g/cm^3^, which was easy to mold, and cure was supplied by the vendor (Kalamazoo, MI).

**Figure 1.**
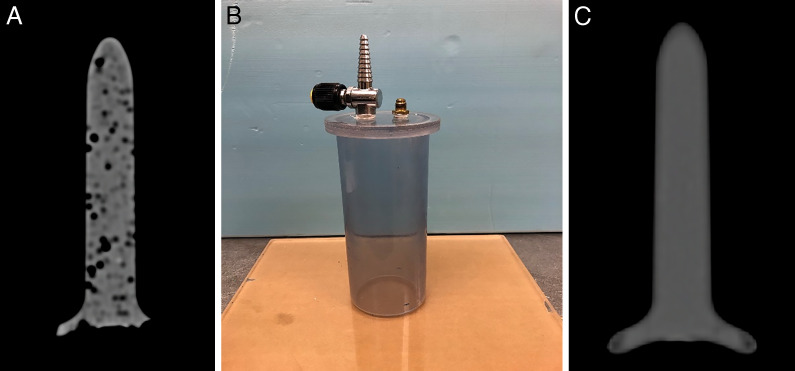
Silicone-filled vaginal spacer with air pockets (A), custom-built vacuum chamber (B), and air-free and silicone-filled vaginal spacer (C).

At the time of initial manufacturing, spacers were filled with the silicone through a small hole that was opened at the bottom of the objects. After the process, the spacers were CT (computer tomography) scanned and revealed numerous air bubbles within the media. To overcome this difficulty, a custom-made vacuum chamber was built to remove air pockets from the spacer as it was filled with the silicone. Using a vacuum chamber was the best method to reduce the amount of trapped gasses, producing a more consistent air-free product. A secondary CT image was taken to verify the homogeneity of the media after the silicone was cured within the spacer. **Figure 1** shows the vaginal spacer and the vacuum chamber, which helped eliminate air pockets that would appear during filling.

### Measurements of Proton Relative Stopping Power for Silicone

**Figure 2.**
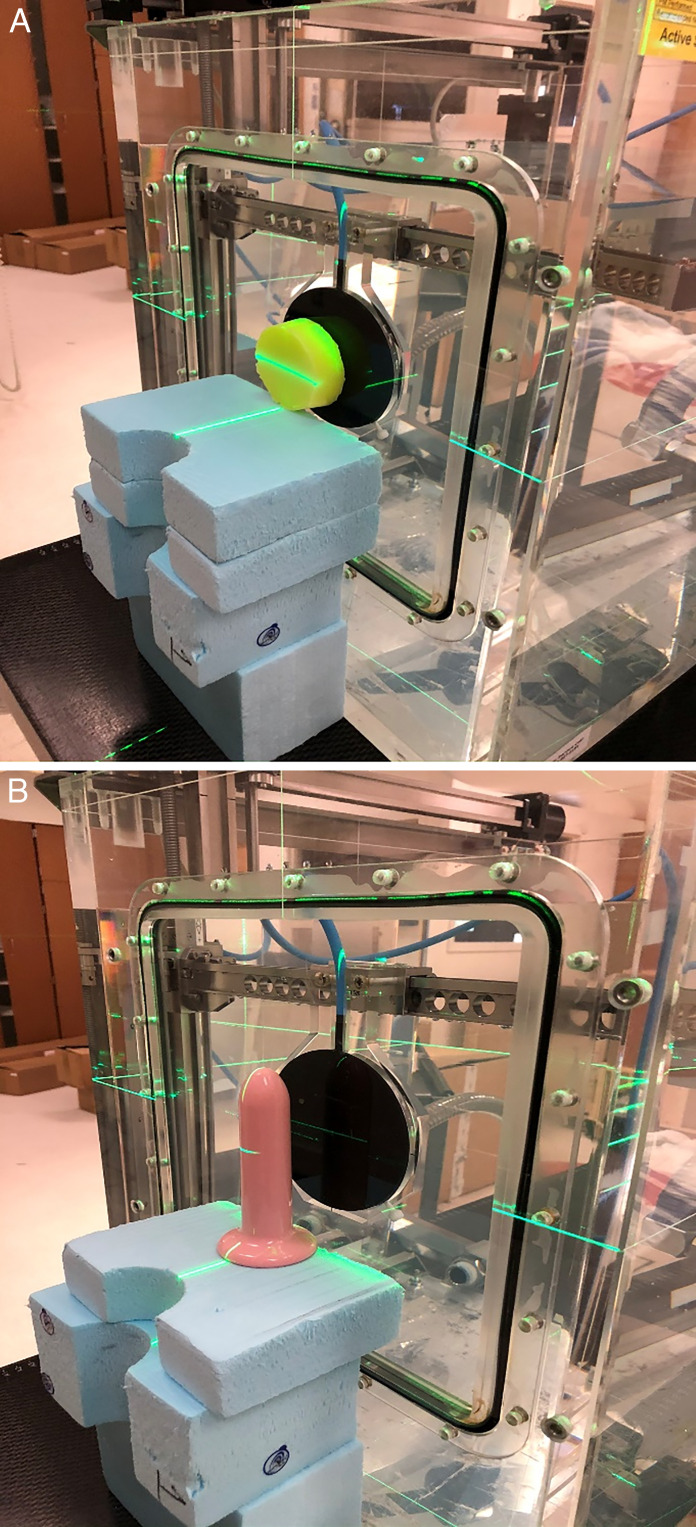
The WET measurements of pure silicone test slab (A), and plastic casing of hollow spacer (B). Abbreviation: WET, water equivalent thickness.

For proton dose calculation, it is crucial to measure proton relative stopping power (pRLSP) of any foreign objects to be used in the treatment planning system (TPS). An experimental setup was prepared for the measurement of proton integral depth-dose curves by using PTW Bragg Peak Ionization Chamber (Freiburg, Germany) to determine the amount of pullback caused by the material in the water tank measurements with 100 MeV of proton beams in a commercial proton beam therapy system (PROBEAT-V, Hitachi America Ltd, White Plains, NY). There were 2 types of materials to characterize in our custom silicone-filled spacer: a hollow plastic outer casing and silicone filling. Therefore, these 2 materials were separately measured in the water tank setup and merged later in the calculation to determine the total water equivalent thicknesses (WETs) of the 5-size vaginal spacers. **Figure 2** demonstrates the 2 different setups for the measurements. In the first setup, a known physical thickness of pure silicone test slab was measured via water tank measurement. In the second setup, only the plastic outer casing with known wall thicknesses was measured to determine the amount of WET in the water tank measurements. These measurements were combined, yielding a sum WET of each spacer (in the maximal cross-sectional dimension) as follows: 1.56 cm, 1.98 cm, 2.63 cm, 3.28 cm, and 3.91 cm for 5 sizes of silicone-filled vaginal spacers.

### Treatment Planning for Pediatric Proton Therapy

Two female patients with rhabdomyosarcoma in the lower pelvis were treated with proton radiation therapy as part of the protocol therapy. Both required delivery of high-radiation doses (59.4 GyRBE) to primary perineal tumors in the perivaginal region. Patient A had a more unilateral tumor with smaller gross tumor volume (GTV; 23.3 cm^3^) irradiated by straight lateral and posterior beams. Patient B had a much larger GTV (342.2 cm^3^) irradiated by 2 posterior oblique beams. Both patients were treated with additional anterior beams for lymph nodes in the inguinal region. The 2 patients received 2 phases of radiation therapy, including phase I (clinical target volume [CTV], prescription dose [Rx] = 36 GyRBE) and phase II (GTV, Rx = 59.4 GyRBE). Vaginal walls were separated and manually drawn in CTs with 4-mm margin each as contralateral and ipsilateral vaginal walls. While the contralateral vaginal wall was directly opposite of the disease site and represents uninvolved vaginal wall, the ipsilateral vaginal wall was situated within the disease site.

**Figure 3.**
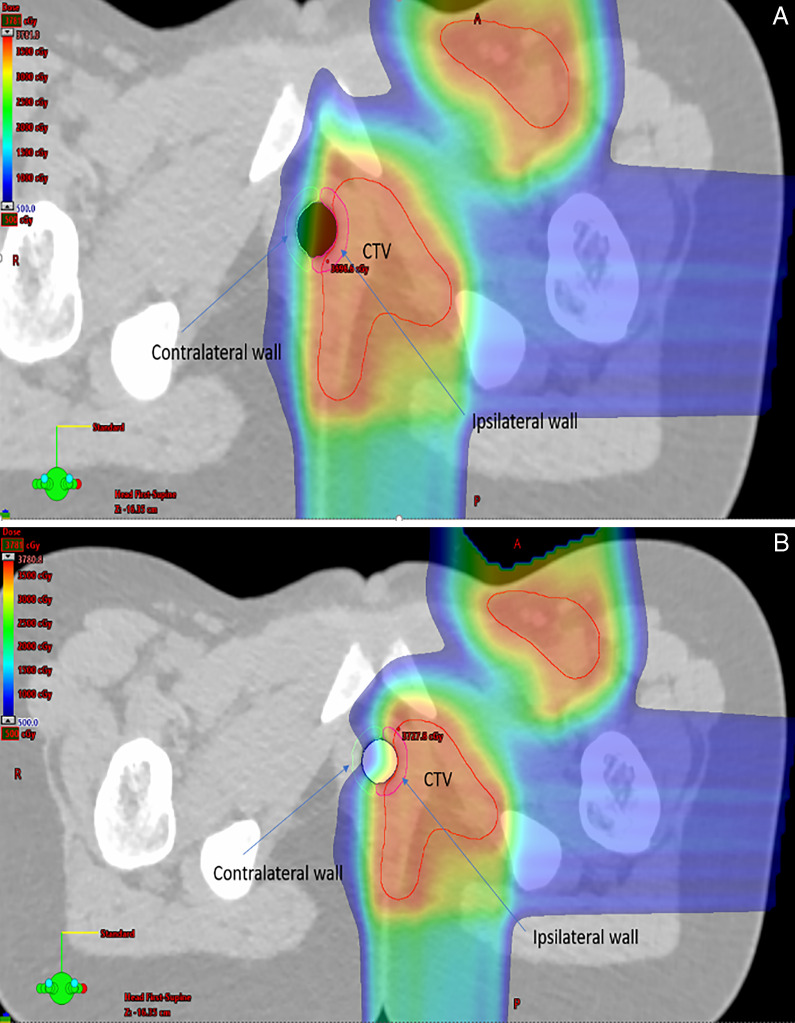
Dose distributions around vaginal wall for hollow vaginal spacer (A) and silicone-filled vaginal spacer (B) with large sparing of contralateral vaginal wall for patient A. Abbreviation: CTV, clinical target volume.

**Figure 4.**
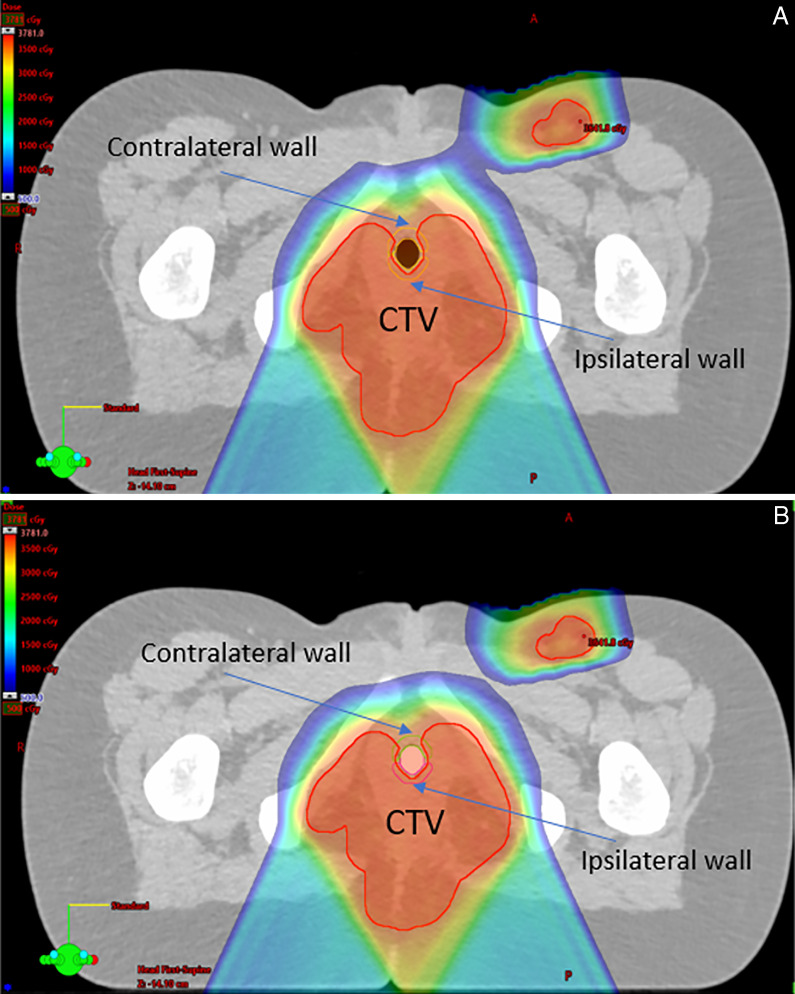
Dose distributions around vaginal wall for hollow vaginal spacer (A) and silicone-filled vaginal spacer (B) with no significant sparing of contralateral vaginal wall for patient B. Abbreviation: CTV, clinical target volume.

During treatment planning, the silicone-filled vaginal spacers were overridden on the planning CT to their correct WET values in Eclipse TPS (Varian Medical Systems, Palo Alto California). For these 2 patients, pRLSPs of the spacers were 1.198. Dosimetric constraints were the same for the 2 patients with a target coverage goal (CTV or GTV) of V95% = 100% of Rx dose and a worst-case scenario of V95% = 95% of the Rx dose including robust optimization of 3 mm in setup and 3% in CT uncertainty. The planning goal for the uninvolved vaginal wall was a mean dose of less than 50% of the Rx dose. The original plans with silicone-filled vaginal spacers were compared to dose re-calculation plans generated from the hollow and no-spacer CTs to quantify the amount of sparing for comparison on the contralateral vaginal wall. **Figure 3** for patient A and **Figure 4** for patient B demonstrate the location of the disease sites and the beam orientations for the 2 patients while there were hollow and silicone-filled vaginal spacers. Contralateral and ipsilateral vaginal walls were also shown to visually inspect the dose perturbations around vaginal walls.

## Results

In the composite plans including 2 phases, the target coverages of the 2 patients were comparable for GTV targets in Dmin, Dmean, and Dmax as 56.7, 58.1, 60.1 GyRBE, respectively, for patient A, and 56.8, 58.2, and 59.8 GyRBE, respectively, for patient B. The dosimetric sparing of contralateral vaginal wall was chiefly achieved in patient A with a mean dose of 8.4 GyRBE when the silicone-filled spacer was used in the actual treatment plan. With the use of only hollow spacer, the contralateral vaginal wall would have received a mean dose of 17.1 GyRBE. In the absence of any spacer, the contralateral vaginal wall would have received a mean dose of 30.8 GyRBE. In patient B, owing to the size and location of the tumor relative to the vagina, the sparing of the contralateral vaginal wall was not observed. The **Table** presents all 3 scenarios, namely with no spacer, commercial hollow spacer, and custom-made silicone-filled vaginal spacer for the 2 patients, including treatment phase 1 and accumulated dose at phase 2.

**Table.** Dosimetric parameters shown for the comparison among 3 scenarios for no vaginal spacer, hollow vaginal spacer, and silicone-filled vaginal spacer for the 2 patients in 2 treatment phases.

**Three dosimetric scenarios**	**Dosimetric parameters, GyRBE**	**Patient A**		**Patient B**	
		**Phase I**	**Phase II**	**Phase I**	**Phase II**
No vaginal spacer	Target CTV or GTV D95	34.9	57.5	34.7	57.6
	Vagina mean dose	23.0	35.2	35.2	58.0
	Ipsilateral vaginal wall mean dose	24.9	38.7	35.2	58.0
	Contralateral vaginal wall mean dose	20.6	30.8	35.2	58.0
Hollow vaginal spacer	Target CTV or GTV D95	34.8	57.4	34.9	57.7
	Vagina mean dose	18.4	28.4	35.2	57.5
	Ipsilateral vaginal wall mean dose	25.0	39.3	35.0	57.6
	Contralateral vaginal wall mean dose	11.6	17.1	35.4	57.5
Silicone-filled vaginal spacer	Target CTV or GTV D95	34.8	57.5	34.9	57.8
	Vagina mean dose	16.2	23.7	35.0	56.7
	Ipsilateral vaginal wall mean dose	24.9	38.6	35.1	57.8
	Contralateral vaginal wall mean dose	7.2	8.4	34.9	55.6

**Abbreviations:** CTV, clinical target volume; GTV, gross tumor volume.

## Discussion

Our study suggests that when the vagina is adjacent but not circumferentially encased with tumor, a significant dose reduction can be achieved with the insertion of a silicon-filled vaginal spacer. Multiple factors need to be taken into account while planning for such cases in which uninvolved vaginal wall is considered as an adjacent organ at risk. These factors can affect the sparing of the contralateral vaginal wall, such as target volume, dimension, location, and shape of the disease site and its proximity to the vaginal walls. Beam orientations and trade-offs between target coverage and organ sparing are also contributing factors to the overall results. Further study is warranted to explore the use of silicone-filled tissue spacers for similar or other applications.
